# Neglected irreducible posterolateral knee dislocation

**DOI:** 10.4103/0019-5413.69323

**Published:** 2010

**Authors:** Raghav Saini, Aditya Krishna Mootha, Vijay G Goni, Mandeep Singh Dhillon

**Affiliations:** Department of Orthopaedics, Postgraduate Institute of Medical Education and Research, Sector 12, Chandigarh – 160 012, India

**Keywords:** Irreducible knee dislocation, knee dislocation, neglected posterolateral knee dislocation

## Abstract

Knee dislocations are rare injuries. Posterolateral knee dislocations are only a small subset of them. There is a paucity of literature regarding the management of such neglected cases. We report here, a case of neglected irreducible posterolateral knee dislocation treated with open reduction and isolated posterior cruciate ligament reconstruction followed by gradual rehabilitation with good outcome at 3 years followup.

## INTRODUCTION

It is unlikely that any single physician personally cares for more than a few knee dislocations in a lifetime of practice is an intercept from a classic article “Traumatic dislocation of the knee joint” by Meyers.^1^ An irreducible knee dislocation is even more rare which is a hallmark of posterolateral knee dislocations as medial femoral condyle buttonholes through the medial capsule and retinaculum.^2^ Treatment protocols for neglected irreducible knee dislocations are not clearly described. We report a 3 months old neglected case of an irreducible posterolateral knee dislocation, for the treatment executed with good outcome.

## CASE REPORT

**Figure 1:**
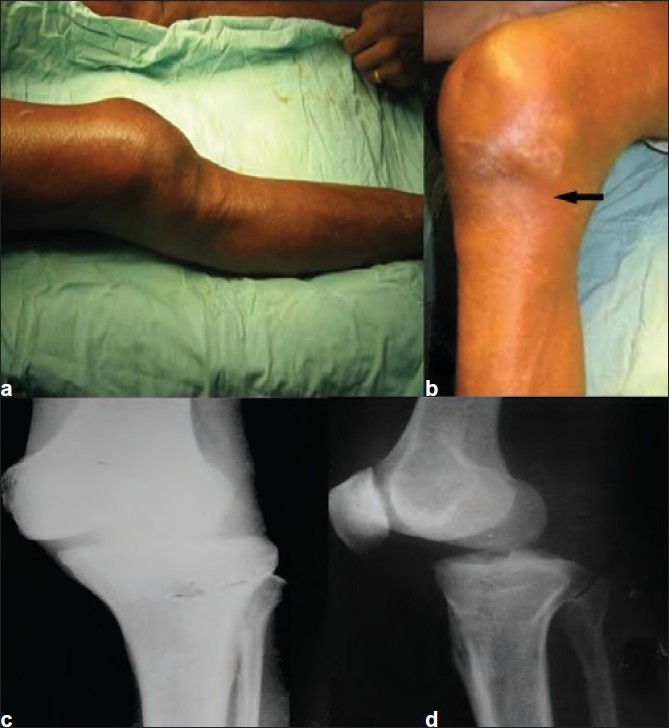
Clinical picture showing (a) the posterior sag and (b) a typical dimple sign (arrow) of the irreducible posterolateral knee joint over the medial joint line. (c) AP and (d) lateral views of the knee joint confirming the posterolateral knee dislocation

A 53-year-old male, pillion rider on a motorcycle had a head-on collision with a car and suffered an injury to the right knee. The patient was treated by a local bone setter in the form of manipulation and immobilization. The patient reported after 3 months of his injury with the complaints of deformity and inability to bear weight. On examination, his knee was locked in 30° of flexion and he could not bear weight on the right lower limb. In addition to the posterior sag of the right tibia [Figure 1a and b], there was associated lateral subluxation of tibia. Typical medial joint line puckering, also called “dimple sign”, was noticed [Figure 1a and b], which accentuated with every attempt of closed reduction.^3^ Based on these findings, a diagnosis of neglected irreducible posterolateral knee dislocation was made and confirmed by radiological examination [Figure 1c and d]. There was no distal neurovascular deficit.

MRI examination of the right knee joint confirmed the mid-substance tear of anterior cruciate ligament (ACL), posterior cruciate ligament (PCL), and medial collateral ligament (MCL). The invagination of the medial capsule and retinaculum by the medial femoral condyle was clearly depicted on MRI.

**Figure 2:**
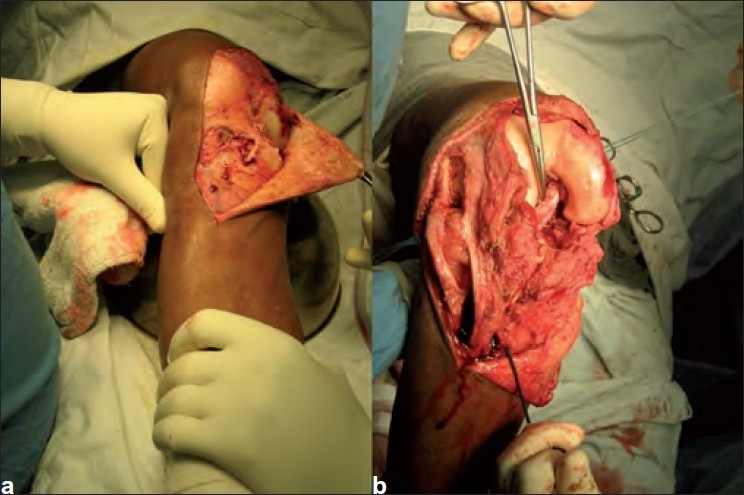
Peroperative photograph showing (a) the medial femoral condyle buttonholes through the medial retinaculum and capsule. (b) PCL reconstruction using the bone patellar tendon bone graft

The Hoppenfeld and de Boer approach^4^ was used for open reduction and PCL reconstruction as same incision could expose medial invaginating structures and the posterior aspect of tibia. The medial femoral condyle was lying just underneath the subcutaneous tissue as it had buttonholed through the medial retinaculum and capsule [Figure 2a]. The medial collateral ligament had a mid-substance tear. Both ACL and PCL were torn and were intermingled with a large amount of fibrous tissue inside the joint leading on to difficulty in dissecting this tissue from the intact medial meniscus. After removing all the invaginated tissue, the knee joint was reduced. Bone patellar tendon bone graft was harvested and PCL reconstruction [Figure 2b] was done using two titanium interference screws. The medial collateral ligament was repaired and augmented with the help of the semitendinosus muscle. The wound was washed and closed in layers and the knee was immobilized with a transarticular unilateral half pin external fixator in 20° of flexion.

On the second postoperative day, blackening was noticed on the medial aspect of the incision in an area of 4 × 3.5 cm. Within a few days, superficial layers of skin were necrosed and sloughed away and the wound healed with a secondary intention within a period of 4 weeks. The external fixator was removed after 6 weeks and knee mobilization was started under supervision with a motion control brace. Partial weight bearing was started at 10 weeks postoperatively with the knee brace locked in extension. For the initial 4 weeks, the patient was allowed toetouch weight bearing, followed by a gradual increase up to 50% by 16 weeks. By 24 weeks, the patient was allowed full weight bearing on the operated side. The knee brace was discarded after 24 weeks. The patient gained flexion of 90° by the end of 6 months. The patient is satisfied with the result and there are no complaints of instability of the joint. The Lachman test and the anterior drawer test were found to be negative, hence ACL reconstruction was not done.

**Figure 3:**
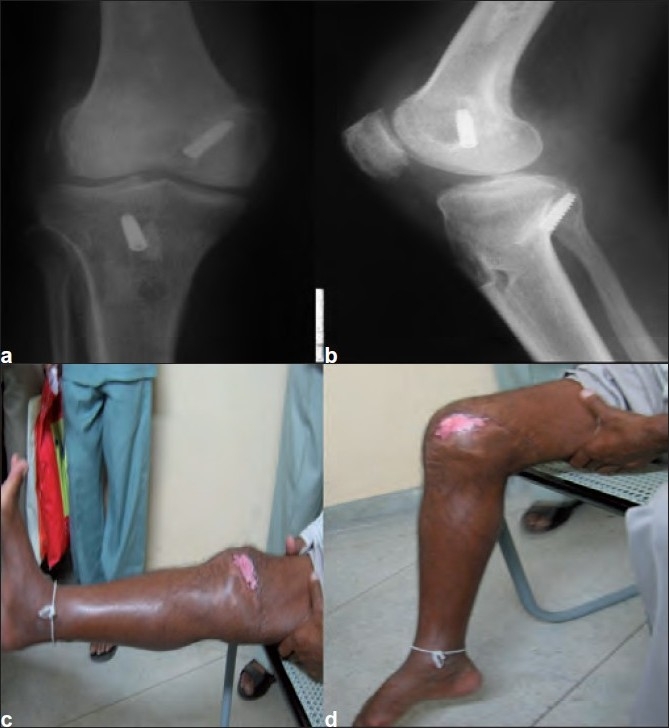
3-year follow-up anteroposterior (a) and lateral (b) views of the knee joint showing the concentric reduction of the joint and no signs of degenerative joint changes. (c and d) 3-year follow-up clinical photographs showing full extension to 90° of flexion with a tell-tale sign of postoperative skin necrosis

At a 3-year follow-up, the patient is walking full weight bearing without a walking aid or brace. The patient does not complain of any pain after 3 years of surgery and follow-up radiographs [Figure 3a and b] also rule out any evidence of degenerative changes in the knee joint. The patient has full extension of the knee joint which can be further flexed to 90° [Figure 3c and d]. Knee Society clinical and functional knee scores at the final follow-up were 88 and 90, respectively.

## DISCUSSION

Knee dislocations are very rare injuries but have some serious neurovascular injuries which might even require amputations.^2^ Most of these injuries could be reduced under sedation by closed methods but a few of them are irreducible and require urgent surgical intervention. Posterolateral knee dislocations are usual culprits in such situations.^5^ There are very few case reports of posterolateral knee dislocations in the literature. The mechanism of injury in such irreducible posterolateral knee dislocation is usually abduction and external rotation forces applied to a flexed knee.^6^ Our patient could not recollect the exact mechanism of injury. In spite of an extensive search of the literature, we were able to find only a few reports of neglected knee dislocations^7^–^10^. Due to rarity of the situation and lack of enough literature, the treatment options for a neglected knee dislocation are confusing. The treatment options ranged from open reduction with ligamentous reconstruction to arthrodesis or arthroplasty.^7^–^10^

Richter *et al*. reported a case of chronic posterior dislocation of knee which was managed by arthrolysis, PCL reconstruction, and a special hinged external fixation device.^8^ In their case, a repeat PCL reconstruction with an Achilles tendon allograft was done due to autograft degeneration after initial surgery. At the final follow-up of 1 year, the patient had painless motion of 50° with mild residual posterior subluxation. Watanabe *et al*. reported a case of chronic knee fracture dislocation combined with popliteal vessel and peroneal nerve injuries treated successfully by the Ilizarov external fixator, tibial plateau osteosynthesis and patellar tendon reconstruction.^9^ However, the case being a fracture dislocation, they achieved a stable knee without any cruciate ligament reconstruction. At the final follow-up of 1 year, the patient had painless flexion of 110° with an extensor lag of 15° and a mild residual posterior subluxation.

Objectives of our treatment were to achieve a painless, mobile, and stable knee joint without much functional limitations. After counseling the patient and keeping in mind the demands of his daily activities, open reduction with ligamentous reconstruction was planned. Considering the principles of management in bicruciate ligament injuries, age, functional demands, need for autografts, and relative high incidence of knee stiffness in simultaneous ACL and PCL reconstruction, we did an open reduction and isolated PCL reconstruction.

Postoperatively, superficial medial skin necrosis complicated the normal wound healing and complete wound healing required a period of 4 weeks without the need of split skin graft or flaps. Contrary to our apprehension, wound healing was complete before 6 weeks and so it did not interrupt the physiotherapy protocol. It is very well documented in the literature that the probability of medial skin necrosis increases as the time interval between the injury and open reduction increases.^2^

In conclusion, open reduction and isolated PCL reconstruction followed by gradual rehabilitation is a valid option for the treatment of a rare condition like a neglected irreducible posterolateral dislocation of the knee.
